# Supplementary material for: Validation of the accuracy of the FAST™ score for detecting patients with at-risk nonalcoholic steatohepatitis (NASH) in a North American cohort and comparison to other non-invasive algorithms
Source: PLoS One. 2022 Apr 15;17(4):e0266859. doi: 10.1371/journal.pone.0266859 (PMC9012361; doi:10.1371/journal.pone.0266859)
Supplement: S1 Table — (DOC) [file pone.0266859.s001.doc]

**Appendix Table 1. % At-risk NASH and FAST** by fibrosis stage

| **Fibrosis Stage** | **N** | **% at-risk NASH** | **Mean (SD)**  **FAST^TM^** |
| --- | --- | --- | --- |
| 0 | 142 | 0% | 0.29 (0.19) |
| 1 | 140 | 0% | 0.44 (0.22) |
| 2 | 120 | 65% | 0.52 (0.23) |
| 3 | 121 | 83% | 0.64 (0.21) |
| 4 | 61 | 57% | 0.68 (0.20) |
| **Total** | **584** | **37%** | **0.49 (0.25)** |
